# Assessing Construct Validity in Math Achievement: An Application of Multilevel Structural Equation Modeling (MSEM)

**DOI:** 10.3389/fpsyg.2018.01451

**Published:** 2018-09-05

**Authors:** Georgios D. Sideridis, Ioannis Tsaousis, Abdullah Al-Sadaawi

**Affiliations:** ^1^Harvard Medical School, Boston Children's Hospital, Boston, MA, United States; ^2^Department of Primary Education, National and Kapodistrian University of Athens, Athens, Greece; ^3^Department of Psychology, University of Crete, Rethymno, Greece; ^4^Department of Psychology, King Saud University, Riyadh, Saudi Arabia; ^5^National Center for Assessment in Higher Education, Riyadh, Saudi Arabia

**Keywords:** multilevel structural equation modeling, nested models, construct validity, multilevel confirmatory factor analysis, level specific misfit, discriminant Validity

## Abstract

The purpose of the present study was to model math achievement at both the person and university levels of the analyses in order to understand the optimal factor structure of math competency. Data involved 2,881 students who took a national mathematics examination as part of their entry at the university public system in Saudi Arabia. Four factors from the National math examination comprised the math achievement measure, namely, numbers and operations, algebra and analysis, geometry and measurement, and, statistics and probabilities. Data were analyzed using the aggregate method and by use of Multilevel Structural Equation Modeling (MSEM). Results indicated that both a unidimensional and a 4-factor correlated model fitted the data equally well using aggregate data, where for reasons of parsimony the unidimensional model was the preferred choice with these data. When modeling data including clustering, results pointed to alternative factor structures at the person and university levels. Thus, a unidimensional model provided the best fit at the University level, whereas a four-factor correlated model was most descriptive for person level data. The optimal simple structure was evaluated using the Ryu and West (2009) methodology for partially saturating the MSEM model and also met criteria for discriminant validation as described in Gorsuch (1983). Furthermore, a university level variable, namely the year of establishment, pointed to the superiority of older institutions with regard to math achievement. It is concluded that ignoring a multilevel structure in the data may result in erroneous conclusions with regard to the optimal factor structure and the tests of structural models following that.

## Introduction

Mathematics achievement is one of the most important criterion to entering college and also on achieving career readiness (Adelman, 2003; Maruyama, 2012), particularly in the fields of STEM (Science, Technology, Engineering, and Mathematics) (Adelman, 1999). Moreover, math achievement (along with verbal skills) is one of the two fundamental sub-components of the widely used conceptualization of academic self-concept (Shavelson et al., 1976; Marsh, 1990; Möller et al., 2009). Researchers have consistently found that math achievement is predicted by both individual and contextual factors. For example, Cvencek et al. (2015) found that students' beliefs about math and their math achievement are linked to their performance, with students with low math efficacy performing lower than students with high math efficacy. Furthermore, gender stereotypes about math achievement (i.e., boys perform better than girls) seem to influence math performance, with girls performing worse than males when their negative gender stereotype is activated (Ambady et al., 2001; Galdi et al., 2014). León et al. (2015), based on Self-Determination Theory (SDT; Deci and Ryan, 1985), reported that autonomous motivation (when students engage in learning from their own choice and preference without external pressure), is positively related to math achievement.

Regarding contextual factors, parental involvement (Kung and Lee, 2016), and family health (Barr, 2015) have been found to be influential factors in predicting students' mathematics achievement. Others have focused on within-school factors such as learning environments and motivational classroom discourse, since they influence the learning process (Vršnik Perše et al., 2010; Herndon and Bembenutty, 2014). The learning environment is the broader context in which the instruction is delivered and is concerned with an institution's policy, curriculum, budget, infrastructure (e.g., libraries, labs, IT facilities, etc.), institutional commitment, quality of academic staff, etc. For example, Gamoran (1992) found that school policies for admitting students to advanced math courses (i.e., standard procedures such as admissions tests vs. nonstandard approaches such as teachers' preferences-perceptions) influences math achievement. Furthermore, previous studies reported that schools located in rural areas compared to schools in urban areas exhibit what is called as the: “rural math achievement gap” (Khattri et al., 1997). Reeves (2015) suggested that one possible explanation for this gap comes from the difficulty of rural schools to attract qualified teaching staff, thereby reducing students' opportunity to practice and master advanced math topics.

In higher education, the foundation year of the university seems to be another important factor which might affect the quality of the offered academic degrees. A recent study by U-Multirank (2014), an organization funded by the European Union to compare university performance across a range of different academic activities, revealed that older universities tend to perform better than newer ones across most measures of research excellence. The term “new university” has been used informally to refer to several different waves of universities created in recent years around the Globe as a result of economic growth in Europe and the US. For example, in the United Kingdom, the term is synonymous *with post-1992 universities* and sometimes *modern universities*, referring to any of the former polytechnics, central institutions or colleges of higher education that were given university status from the British government post 1992.

In a study among academic staff at UK universities, it was reported that 75% of respondents in the old universities in UK were located in departments ranked “4” and above (indicating an outstanding performance), whilst no respondents from newer universities found themselves in departments ranked higher than a “3A” (indicating a moderate performance, see Harley (2002). More recently, recent higher institution evaluations in the U.K. showed that older, compared to younger establishments had higher student entry standards, graduate prospects, research quality and intensity, smaller staff-to-student ratios, better facilities, larger amounts of time spent on academic services, receipt of honors from students, and increased rates of degree completion (University League Tables and Rankings, 2017). In a recent study, McCormack et al. (2014) examining more than 250 university department across 100+ UK universities in terms of management practices -shown to predict academic excellence-, found that departments in older universities tend to be better managed than departments in newer universities.

Several reasons for the superiority of the old established universities compared to new ones have been proposed, including a more robust organizational structure (due to tradition), a better infrastructure (e.g., libraries, labs, IT facilities, etc.), more qualified staff (higher academic achievements), better networking after graduation (providing better employment prospects), and a better academic environment (e.g., clubs, social events, sports, etc.). The above factors likely contribute to a differential attraction by older institutions of more qualified individuals, that also result in higher graduation rates and better qualified professionals (Aghion et al., 2010). One important hypothesis of the present study was to test math achievement level differences between old and new establishments, after concluding the optimal factor structure at the university level of the analysis.

Up until recently, the measurement of complex constructs and competencies involved data at the individual level. More recently, however, the advancement of Structural Equation Modeling (SEM) expanded our previous use of data at only a single level in the analysis, namely the person level, though accounting for data complexities and higher order relations (Brown, 2015). Several researchers (McDonald and Goldstein, 1989; Longford and Muthén, 1992) have proposed that patterns of relationships between variables may be different when taking into account nesting in that a relationship between two constructs or two measurements maybe saliently different when viewed under the lenses of a person level analysis (e.g., when the unit analysis is individuals) versus a cluster level analysis (e.g., when the unit of analysis is a cluster of individuals,—e.g., an organization). The combination of the two has contributed to what is now known as Multilevel Structural Equation Modeling (MSEM) which essentially combines the methodologies of structural equation modeling and multilevel modeling (Rabe-Hesketh et al., 2004; Heck and Thomas, 2009). At the measurement level in the analysis, this combination may suggest that different simple structures (i.e., factor solutions) may be operative at different levels in the analysis. At the structural level one can predict different outcome variables that emerge from the earlier measurement models and posit structural paths at each level in the analysis. For example, in measuring academic achievement it is possible that a 5-domain factor structure (e.g., math, language, biology, chemistry, social studies) best describes individuals (who due to “domain specificity” may have variable performance across subject matters), but it is possible that a one-factor structure is the most parsimonious solution at the university level, with good universities having higher levels of achievement *across* subject matters. The implications for including nesting in our evaluation of measures is tremendous for construct validation as a given instrument may operationally define differently a construct at one level in the analysis (e.g., student with a 5-factor solution) compared to another level (e.g., university level where a general achievement factor best fits the data). Such findings have implications not only for theory development and falsification but also, measurement, which lies in the core of all scientific efforts. That is, if proper levels of measurement error are at the person level, then scores are valuable and interpretable; If not, unreliable and likely detrimental for accuracy and prediction. Furthermore, the constructs under study may have different interpretations at each level in the analysis with implications for both operational definitions and use of scores (Huang et al., 2015). Up until recently, the only available means for evaluating the construct validity of instruments confounded (through ignoring) the presence, and differential effects, that nesting of individuals within clusters may exert on the data. The purpose of the present study was to demonstrate, using a national measure of math achievement, the evaluation of its optimal factor structure through accounting for the correlated structure of the university where students originate from using Multilevel Structural Equation Modeling (MSEM).

### Multilevel structural equation modeling (MSEM)

Multilevel Structural Equation Modeling (MSEM) evaluates measurement and structural models at more than one levels in the analysis when nesting is in place (Geldof et al., 2014; Heck and Thomas, 2015). The primary purpose of modeling data at two or more levels is to avoid the violation of the independence of observations assumption which is introduced when ignoring the clustering variable (e.g., the effects a school administration, teacher, school culture, or classroom climate exerts on all students-causing a baseline between person correlation that reflects a systematic source of measurement error) (Julian, 2001). That is, participants within a cluster are expected to have a higher correlation compared to individuals between clusters (e.g., within a class versus between classes). As shown earlier, such factors have proved to influence math achievement in significant ways (Vršnik Perše et al., 2010; Herndon and Bembenutty, 2014), thus it is important to examine their influential role within a multilevel perspective.

In the present study, we employed MSEM as a means of evaluating the math achievement at both the person and university levels of the analysis. The hypothesis of testing a simple factor structure at the person level of analysis makes inherent sense and is linked to assessment and evaluation, using person scores for future decision making, etc. However, the idea of testing math achievement at the university level of analysis needs to be justified (as well as for any other clustering variable for that purpose). At the measurement level in the analysis, universities are evaluated for the quality and standards they provide to their students, and that evaluation is oftentimes a function of their students' performance. Furthermore, within a university different emphasis may exist that are associated with differential levels of performance. Thus, to evaluate the role different universities might play on math achievement performance, one needs to test the most optimal measurement model for the assessment of a particular domain (e.g., math) in order to make informed decisions (such as staff recruitment, proper allocation of funds, and future university planning) based on that measurement model. For example, in the U.S. in 2013 most of the Federal and State funding was directed to community colleges and small universities compared to research institutions which focused on research grant funding (Woodhouse, 2015). Such decisions need to be grounded in empirical evidence in order to evaluate the services and qualities provided by small and newer institutions compared to older and larger research universities. Furthermore, when involving the MSEM methodology, one is also able to predict students' achievement from university and department level variables such as the year an establishment became a higher education institution, ratio of students to staff, facilities, etc.

If person level math achievement and university level math achievement do not match, measurement—wise, then the most optimal factor structure at each level in the analysis needs to be estimated and applied. The equivocal assumption that person level and university level achievement match, is clearly a tentative assumption and needs to be empirically tested. Based on the above, the purpose of the present study was to model math achievement at both the person and university levels of the analyses to understand the optimal factor structure of math achievement using information from the factor model at each level in the analysis and test the invariance of the proposed structure at the person level by gender. A secondary goal was to predict math achievement at different levels in the analysis, after estimating first the most optimal factor structure.

## Materials and methods

### Participants

Participants were 2,881 individuals who took the math teacher test during a national examination at the National Center for Assessment in Higher Education in Saudi Arabia. The participants took on the measure as part of a licensure program to teach mathematics in elementary and higher education. There were 1,672 males and 1,209 females. The mean age was 24.02 years with an S.D. of 2.517 years (Males: Mean = 23.28, *SD* = 1.837; Females: Mean = 25.35, *SD* = 3.586). Participants were nested within 22 universities, which were classified as “new” if they were established within the last 10 years, or “old” establishments. Consequently, 511 students were nested within “oxsld” universities and 2,370 within “new” universities.

### Measure

#### Math achievement

The present measure was a standardized math competency examination, which was administered regularly as part of satisfying requirements for licensure by the state in Saudi Arabia, thus, they were part of a National Examination study. The instrument included four subscales, namely: (a) numbers and operations (6 exercises), (b) algebra and analysis (17 exercises), (c) geometry and measurement (13 exercises), and (d) statistics and probabilities (7 exercises). Exercises were administered using standardized instructions using a paper-and-pencil format within a specific time period (30 min per domain) and were scored as either correct or wrong. The instrument We opted for creating item parcels^1^ because models based on parceled data: (a) are more parsimonious, (b) present heightened reliability, (c) have distributions that approximate normality, (d) have fewer chances for residuals to be correlated or dual loadings to emerge (both because fewer indicators are used and because unique variances are fewer), and, (e) are associated with enhanced model fit (Bagozzi and Heatherton, 1994; Marsh et al., 1998; Bandalos and Finney, 2001). Furthermore, one of the main weaknesses of item-level factor analysis (i.e., the assumption that the observed variables are continuously measured interval-level data) may be partially overcome using item parcels (Panter et al., 1997). Parcels were created using 3–4 exercises per parcel selected at random from the domain's exercise pool, in order to account for systematic measurement error due to serial dependency, level of difficulty, or content similarity. Consequently, data were analyzed by use of Maximum Likelihood as recommended in the literature when data have 3 or more categories (Dolan, 1994; Beauducel and Herzberg, 2006). A prerequisite assumption to utilizing parcels, however, pertained to observing normality of the parcels' distributions, which was evaluated through inspecting values of skewness and kurtosis. We differed from utilizing the K-S statistic, as using our large sample size, trivial deviations from normality would likely support alternative model hypotheses. For skeweness and kurtosis acceptable values have been reported in the range of ±2 (Field, 2000, 2009; Trochim and Donnelly, 2006; Gravetter and Wallnau, 2014) or ±1.5 (Tabachnick and Fidell, 2013). In the present study, values of skeweness ranged between 0.061 and 1.297 and kurtosis between −0.745 and +0.708, all laying within acceptable limits.

### Data analyses

Data were analyzed using Multilevel Structural Equation (MSEM). Initially, a series of confirmatory factor analysis (CFA) models were tested to verify the proper simple structure using aggregate data, ignoring nesting. We tested a model consisting of four latent variables (Numbers/Operations, Algebra/Analysis, Geometry/Measurement, and Statistics/Probabilities) using item parcels as indicators per latent variable. Structural Equation Modeling (SEM) evaluates discrepancies between data based and hypothesized variance-covariance matrices by use of an omnibus chi-square test using a system of linear equations. Provided that the chi-square test is a test of “exact fit” and thus, any model with measurement error is bound to be rejected as misfitting the data, a number of ancillary descriptive fit indices are oftentimes employed, along with residual values. Specifically, fit indices such as the Comparative Fit Index (CFI) as an absolute fit index^2^, the Tucker-Lewis index (TLI) as an incremental fit index^3^, and unstandardized residual values (Root Mean Square Error of Approximation) need to be greater than 0.900 and less than 0.08, respectively, to suggest a strong resemblance between sample-based and hypothesized variance-covariance matrices. These indices were utilized with both aggregate and multilevel data as there are currently no level specific fit indices (with the exception of the SRMR) that are available in commercial programs. Ryu and West described how to estimate CFI and RMSEA values for their partially saturated approach, but these methods are not currently available in any software as there is no direct estimation of the independence model for each level of the analysis^4^. Last, information criteria in the form of the Akaike index were employed using difference values using conventions described by Raftery (1995).

At a second step in the analysis, the simple factor structures were tested for verification at both levels in the MSEM analysis (person-within and university-between levels in the analyses) assuming there were ample levels of variance at the clustering level (prerequisite assumption). Data were analyzed by means of Maximum Likelihood (ML) estimation, which results in inflated estimates when residual observations are correlated (Pornprasertmanit et al., 2014). Multilevel Structural Equation Modeling (MSEM) involves random variation due to individual differences (individual-within level) and random variation due to groups in which the individuals belong to (group-between level) with the response of person i who belongs to group j on item y being a function of the between-group random component (y_Bj_) and the within-group random component (y_Wij_) as follows (Ryu and West, 2009):

(1) $$\begin{array}{r} y_{ij}=y_{Bj}+y_{Wij} \end{array}$$

With the individuals who belong to the same group having an enhanced relationship compared to individuals belonging to different groups. As the total variance-covariance matrix is decomposed to within and between levels, these components are orthogonal. Since, theoretically speaking the mathematics measure was designed to assess four domains, the estimation of a 4-factor simple structure at the within and between levels was estimated using the Equations (2) through (4) as described by Muthén and Asparouhov (2009), using slightly different notation:

(2) $$\begin{array}{r} y_{ij}=\Lambda_{j}\eta_{ij} \end{array}$$

With subscripts *i* and *j* being the person and clustering units, respectively. In Equation (2), *y*_*ij*_ is a vector of measured variables and Λ_j_ a matrix of factor loadings linking the measured variables to corresponding latent variables at both the within and between levels of the analysis. Subsequently, the within part of the model is estimated as follows:

(3) $$\begin{array}{r} \eta_{ij}=\alpha_{j}+B_{j}\eta_{ij}+\zeta_{ij} \end{array}$$

The above equation involves estimating the within level model of the latent responses *Y*_*ij*_ as being part of a common factor model that includes random intercepts α that vary over clusters *j*. *B*_*j*_ contains a matrix of factor loadings at the within levels and ζ_ik_, residual values of the unique and common factor model at the within level. The between, structural, part of the model is estimated using the following formula:

(4) $$\begin{array}{r} \eta_{j}=\mu +\beta_{j}\eta_{ij}+\zeta_{j} \end{array}$$

**Figure 1:**
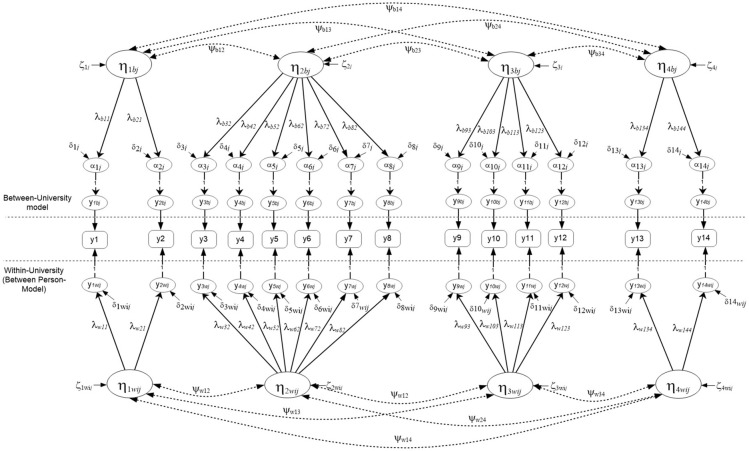
Multilevel Structural Equation Model Positing a 4-factor correlated solution at both the within and between levels of the analysis (*N* = 2,881).

Which contain all random coefficients of intercepts α and slopes B, that vary over clusters *j*, the means μ and the factor loadings estimated from the between-cluster variance-covariance matrix β. Last, ζ_k_ contains residual values of unique and common factors at the between level of the analysis. Subsequently, the hypothesized 4-factor correlated model that describes math competency as a 4-dimension correlated structure (see Figure 1) at both levels of the analysis is shown below using expanded matrices^5^ (see also Geldof et al., 2014 for an explanation of the notation). Multilevel Structural Equation Model (MSEM) using matrix notation that posits a 4-factor structure at both the within and between levels of the analysis.


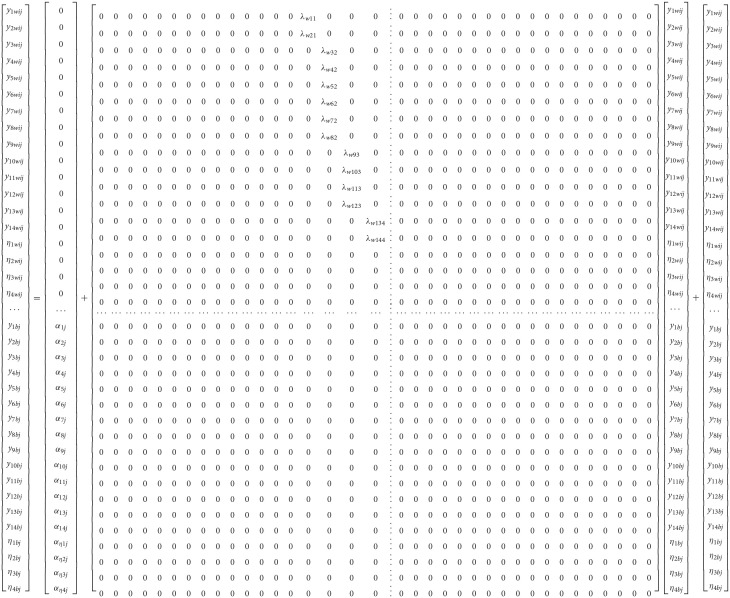



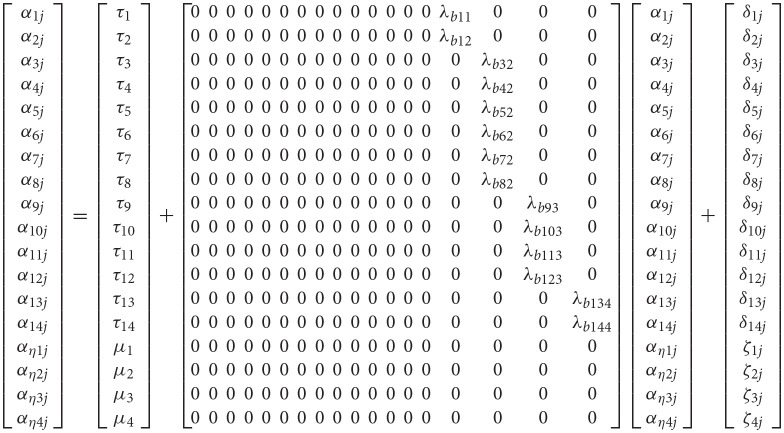


Figure 1 shows the hypothesized 4-factor structure. Note that with the above notation of Equations (2–4), all variables are treated as endogenous (i.e., dependent). For alternative conceptualizations see Muthén and Asparouhov (2009). Also, factor loadings were not fixed to unity; instead, identification of the metric of the factor should be done by fixing the factor variance to unity when modeling the data.

The test of simple factor structures involved several stages. At a first stage the optimal factor structure at a specific level in the analysis (e.g., multi-factor correlated) was contrasted to a competing structure (e.g., unidimensional), after saturating the other level of the analysis. The goal of this test was to conclude the optimal structure at each level in the analysis controlling for measurement error introduced by the structure tested at the other level of the analysis. This *partially saturated* modeling approach was first introduced by Hox (2002) and then expanded by Ryu and West (2009). It provides a test of model fit at each level in the analysis, as currently there are no level-specific fit indices to evaluate model misfit (except for the SRMR index). The standard method for evaluating model fit in SEM involves the use of a likelihood ratio statistic that tests the null hypothesis^6^ that data-based model fit, as estimated using the ML fitting function, is equivalent to the fit of a saturated model and, thus, there is no difference between the data-based model and a “perfectly fitted model.”:

(5) $$\begin{array}{r} T_{ML}=F_{ML}\left(\overset{´}{\theta }\right)-F_{ML}\left({\overset{´}{\theta }}_{s}\right) \end{array}$$

Applying the standard approach (Yuan and Bentler, 2007) to nested structures would suggest that overall model fit would be dominated by the within group model potential misfit for which there is a larger sample size and thus, overall model fit woucertainly downplay misfit due to the between level of the analysis. This is due to the fact that the entire model is estimated simultaneously to test for the goodness of fit of a model (i.e., both covariance components at the between level Σθb and the within level Σθw). In other words, a conclusion pointing to a misfitted model would fail to describe the location of the misfit. Ryu and West (2009) proposed a partially saturated model fit approach in which the discrepancy between estimated and saturated models would be restricted to one level in the analysis. For example, if one wants to test a within group model with no misfit introduced by the between group model the later has to be saturated. A chi-square test would then test the discrepancy due to the within level as shown in the equation below:

(6) $$\begin{array}{r} \chi^{2}=F_{ML}\left[\Sigma_{b}\left({\overset{´}{\overset{´}{\Theta }}}_{S}\right),\;\Sigma_{W}\left(\Theta \right)\right]-F_{ML}\left[\Sigma_{b}\left(\Theta_{S}\right),\;\Sigma_{W}\left({\overset{´}{\Theta }}_{S}\right)\right] \end{array}$$

With any potential misfit found above attributed to discrepancies between estimated and saturated within level covariance matrices only. Consequently, we adopted this approach to test level specific model fit.

At a second stage, we tested the discriminant validity of the simple structure's components via the Gorsuch (1983) approach, which involves contrasting a model where between factor covariations are freely estimated to a model that these covariations are constrained to be equal to 1. If the later model is not inferior to the freely estimated model, then the hypothesis that the factors assess conceptually distinct components is not supported (and thus, unidimensionality is the likely alternative). Consequently, the proposed simple structure should fit the data well at both levels of analysis and should meet criteria for discriminant validation, when appropriate.

At a third step in the present modeling, the functioning of gender was investigated as a within person variable for which the proposed factorial structure may not hold (measurement non-invariance). Thus, a series of Differential Item Functioning (DIF) analyses were conducted by use of the Multiple Indicators Multiple Causes (MIMIC) model (Muthén, 1978; Mislevy, 1986) following the Muthén (1989a) approach. The model tests the probability that item u_j_ that belongs to factor η_i_ and receives a direct effect from a dichotomous covariate x_i_ (gender in the present case) has a response probability of 1 as shown below (Gallo et al., 1994):

(7) $$\begin{array}{r} u_{ij}=\lambda_{j}*\eta_{i}+\kappa_{j}*x_{i}+\varepsilon_{ij} \end{array}$$

with λ being the factor loading of item *u*_*j*_ on factor η with a mean of zero, κ_*j*_ being the effect of the covariate on item *u*_*j*_ at values *x*_*i*_. The probability of correct responding is then estimated as follows:

(8) $$P\left(u_{ij}=1\;|\eta_{i},x_{i}\right)=1-F\left[\tau_{j}-\lambda_{j}*\eta_{i}-\kappa_{j}*x_{i}\right)*\theta_{jj}^{-1/2}],$$

With θ_*jj*_ being the item residual variance, τ_*j*_ the item threshold, λ_*j*_ the factor loading, η_*i*_ the factor mean (usually specified to be zero), κ_*j*_ the effect of the covariate on item *j*, and *F* the normal distribution function (Muthén, 1989a,b). The approach utilized herein for testing invariance across gender at the person level in the analysis has been described by Muthén (1989a,b) and involves the following steps: (a) test for optimal factor structure, (b) test for effects of covariate(s) through constraining those direct effects to zero and evaluate magnitude of modification indices, verifying that factor structure does not change, (c) add direct effects of covariate on items recommended by modification indices, verifying again that the factor structure does not change as a function of modeling the covariate, (d) conclude on meeting requirements for full or partial invariance due to the covariate. All analyses were run using Mplus and Maximum Likelihood (ML) estimation using raw data as inputs and through analyzing variance-covariance matrices.

## Results

### Simple structure of math test overall^7^

**Figure 2:**
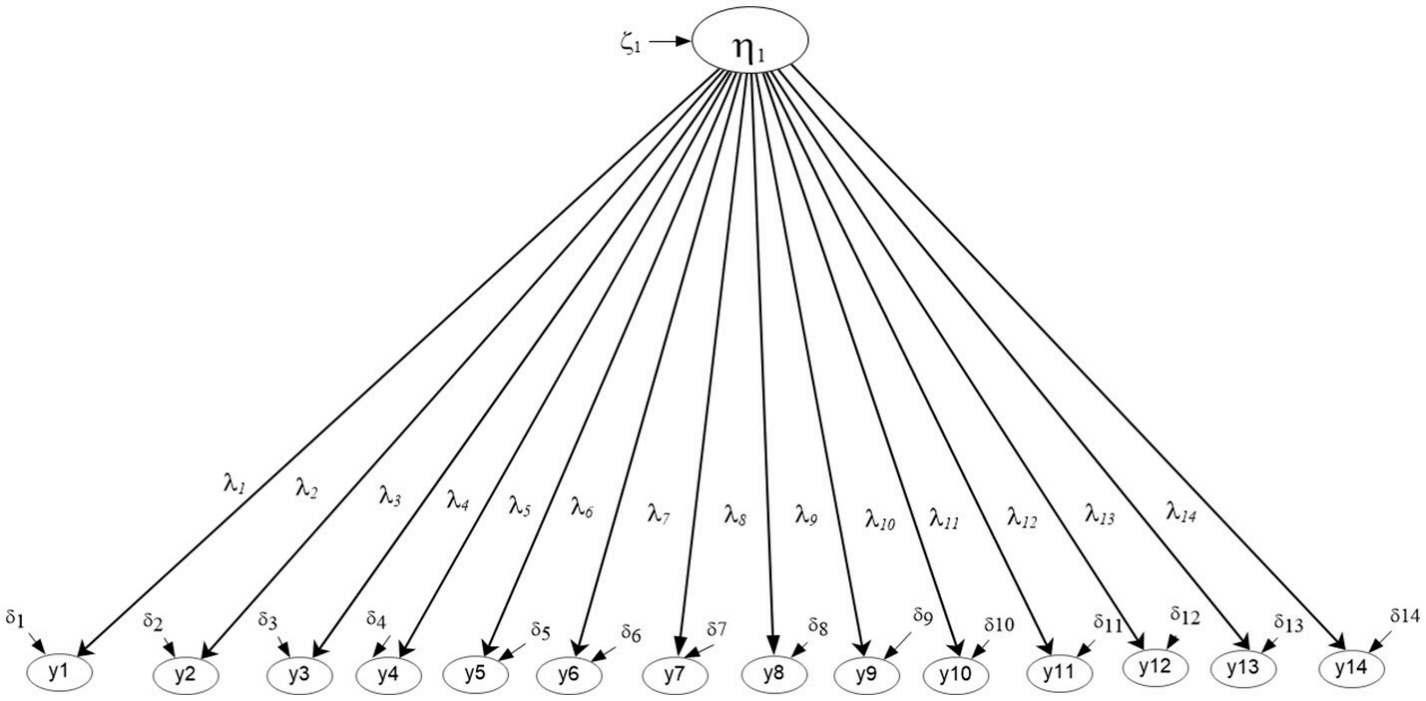
Unidimensional Mathematics Competency model tested with aggregate data based on parceled items (*N* = 2,881).

**Figure 3:**
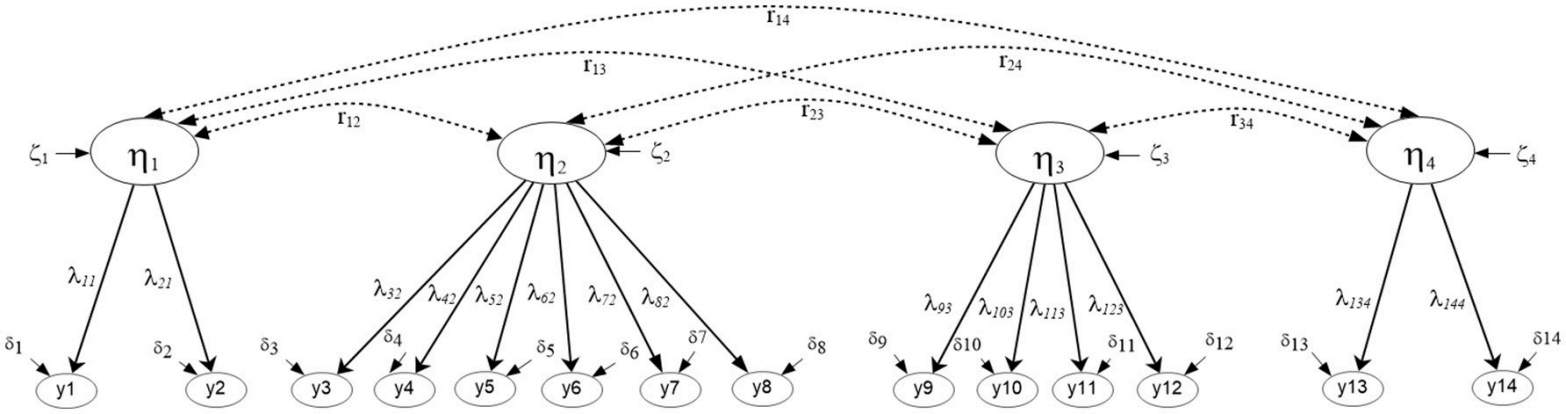
Four-factor correlated model tested with aggregate data based on parceled items. The latent mathematics factors were: η_1_, Numbers/Operations; η_2_, Algebra/Analysis; η_3_, Geometry/Measurement; η_4_, Statistics/Probabilities. (*N* = 2,881).

Figures 2, 3 display the simple structures tested with aggregate data (i.e., ignoring clustering due to university) including a unidimensional, and a multidimensional model. Results indicated that model fit was adequate using unstandardized residuals (RMSEA) and less so the descriptive fit indices across all models (e.g., Unidimensional Model RMSEA = 0.013, CFI = 0.985; Multi-factor model RMSEA = 0.011, CFI = 0.985). When comparing the models using a Chi-square difference test, model fit was not significantly different between the two nested models, the univariate and multi-factor, although the multi-factor model showed slightly better fit [Δ_Chi−square(6)_ = 8.119, *p* > 0.05] (see Table 1). By use of the AIC and BIC parsimonious indices the univariate model was deemed the preferred model for these data (Univariate AIC = 185,519.350; Multi-factor AIC = 185,523.231; Univariate BIC = 185,796.653; Multi-factor BIC = 185,840.149), although effect size indicators of the AIC (Raftery, 1995) suggested that whenever the difference in AIC values is less than 10 units (3.881 in the present instance), there is not *strong* support for the superiority of one model over another. Interestingly, this early conclusion was severely challenged in the next section, after modeling the mathematics simple structure at each level in the analysis and after testing misfit at each level of the analysis using the Ryu and West methodology.

**Table 1:** Comparison of simple structures of math achievement using aggregate data.

	**Model Chi-square**	**Degrees of freedom**	**Δ-Chi-square**	**Δ-Degrees of freedom**	***p*-value**
M1. Unidimensional Simple Structure	145.141*	77	–	–	–
M2. Four-factor Correlated Model	137.022*	71	8.119	6	n.s.

* *p < 0.01; The level of significance was set to 0.01 to adjust for the excessive levels of power associated with an n-size of 5,445 participants. The critical value of a Chi-square statistic with 3 degrees of freedom is 11.345 at p < 0.01*.

*n.s. = Non-significant*.

### Simple structure of MA at the between-person (within) and between-university (between) level

A series of models were fit to the data and subsequently compared and contrasted in order to determine the best fitted model at each level in the analysis (person or university). However, it was necessary to first test that variability in math achievement scores was present at the university level of analysis (Raudenbush and Bryk, 2002; Maas and Hox, 2005). Consequently, a series of Intraclass Correlation Coefficients (ICCs) were assessed in order to verify that variances of the match exercises at the between-university level were non-zero (Werts et al., 1974; Raykov, 1997; Hsu et al., 2016). The coefficient is estimated as the ratio of the between-level variance ${\text{σ}}_{\text{u}\text{0}}^{\text{2}}$ to that of the total variance (within ${\text{σ}}_{\text{r}}^{\text{2}}$ and between ${\text{σ}}_{\text{u}\text{0}}^{\text{2}}$) and makes use of the null model as follows (Kreft and de Leeuw, 2004):

(9) $$\begin{array}{r} ICC=\frac{\sigma_{u0}^{2}}{\left(\sigma_{u0}^{2}+\sigma_{r}^{2}\right)} \end{array}$$

with $\sigma_{u0}^{2}$ being the cluster-based variance and $\sigma_{r}^{2}$ the between-person within cluster variability. Furthermore, we supplemented the ICC analysis using the “design effect” index (Muthén and Satorra, 1995) which targets at correcting the negative bias associated with nested data due to the violation of the independence of standard errors. It contributes a multiplier that intents to correct standard errors. It is computed as follows:

(10) $$\begin{array}{r} \text{DesignEffect}=1+\left(n_{\text{c}}-1\right)*\text{ICC} \end{array}$$

with n_c_ being the number of level-1 units that comprise the clustering variable. As shown in the above equation the design effect is a function of both the number of units in the clustering variable but also the magnitude of the ICC. Values that warrant the need to account for the correlated structure due to clustering are in excess of 2.0 units. Table 2 shows those estimates which confirmed the need to model the information at the university level of the analysis.^9^ Table 3 provides significance tests based on difference chi-square test statistics for nested models. The models tested were ordered based on the number of modeled parameters (from parsimonious to more parameterized) and were: (a) a one-factor model at both levels, (b) a unidimensional model within and multidimensional between, (c) a multi-factor model within and one-factor model between, and, (d) a multi-factor model at both levels^10^. Of interest was the comparison between unidimensional and multi-factor structures at both levels in the analyses, in light of the fact that there was no significant difference between the univariate and 4-factor correlated model with the aggregate data (i.e., when ignoring the nesting of participants onto clusters).

**Table 2:** Intraclass Correlation Coefficients (ICCs) of math items along with 95% confidence intervals, tests of significance and design effect values.

**Math exercises**	**ICC (%)**	**95% Confidence interval**	***Z*-Test**	***p*-value**	**DEFF**
Number and Operations 1	1.9	0.001 to 0.036	2.121	0.034*	5.921
Number and Operations 2	5.7	0.019 to 0.095	2.927	0.003**	15.763
Algebra and Analysis 1	2.4	0.005 to 0.042	2.551	0.011*	7.216
Algebra and Analysis 2	3.7	0.010 to 0.065	2.639	0.008**	10.583
Algebra and Analysis 3	1.4	0.001 to 0.027	2.129	0.033*	4.626
Algebra and Analysis 4	0.3	−0.003 to 0.010	1.023	0.306	1.777
Algebra and Analysis 5	4.9	0.016 to 0.083	2.875	0.004**	13.691
Algebra and Analysis 6	0.6	−0.001 to 0.013	1.778	0.075†	2.554
Geometry and Measurement 1	0.8	0.000 to 0.017	2.035	0.042*	3.072
Geometry and Measurement 2	4.2	0.013 to 0.071	2.825	0.005**	11.878
Geometry and Measurement 3	4.2	0.013 to 0.071	2.825	0.005**	11.878
Geometry and Measurement 4	1.7	0.002 to 0.032	2.230	0.026*	5.403
Statistics and Probabilities 1	5.2	0.017 to 0.088	2.902	0.004**	14.468
Statistics and Probabilities 2	4.1	0.011 to 0.071	2.648	0.008**	11.619

*The above ICCs may appear on the low side but, although not customary, tests of significance and confidence intervals were constructed based on the parametric bootstrap distribution using routines initially developed for use with categorical data (Preacher and Selig, 2012; Raykov and Marcoulides, 2015,^8^). As shown above only the Algebra and Analysis exercises 4 and 6 did not present themselves with ample variability at the university level of the analysis. Further information was provided by use of the Design Effect (DEFF) index for which values greater than 2.0 suggest the need to employ a multilevel structure*.

* p < 0.05;

** *p < 0.01*.

*Significance using a one-tailed test at p < 0.05*.

**Table 3:** Comparison of simple structures of math achievement across levels in the multilevel structural equation modeling (MSEM) analysis.

**Model comparison**	**Chi-square**	**D.F**.	Δ-Chi-square*,^†^	**Δ-D.F**.	**Δ-Sig**.	**AIC**	**Δ-AIC**	**BIC**	**Δ-BIC**	**SABIC**	**Δ-SABIC**	**CFI**	**RMSEA**
1W 1B	190.357	154			<0.050	185,062.950	–	185,518.519	–	185,299.259	–	0.992	0.009
1W 4B	185.269	148			<0.050	185,072.927	–	185,568.111	–	185,329.784	–	0.992	0.009
4W 1B	130.885	148			<0.050	185,066.877	–	185,562.060	–	185,323.734	–	1.00	<0.001
4W 4B	126.903	142			<0.050	185,076.955	–	185,611.754	–	185,354.362	–	1.00	<0.001
1W 4B vs. 1W 1B	185.269	148	5.088	6	0.533	–	−9.977	–	−49.592	–	−30.525	–	–
4W 1B vs. 1W 1B	130.885	148	59.472	6	<0.001	–	−3.927	–	−43.541	–	−24.475	–	–
4W 4B vs. 1W 1B	126.903	142	63.454	12	<0.001	–	−14.005	–	−93.235	–	−55.103	–	–
1W 4B vs. 4W 1B	185.269	148	n.a.	0	n.a.	–		–		–		–	–
1W 4B vs. 4W 4B	185.269	148	58.366	6	<0.001	–	−4.028	–	−43.643	–	−24.578	–	–
4W 1B vs. 4W 4B	130.885	148	3.982	6	0.679	–	−10.078	–	−49.694	–	−30.628	–	–

* *p <0.01; The level of significance was set to 0.01 in order to adjust for the excessive levels of power associated with an n-size of 2,881 participants*.

n.s., Non-significant finding using an alpha level of 0.01.

† It may sound strange that negative chi-square values are associated with tests of significance. Absolute chi-square values were utilized in those instances as it is possible that modeling additional parameters was associated with decrements in model fit, which was the case moving from the 1W4B to the 4W4B model.

AIC, Akaike Information Criterion; BIC, Bayesian Information Criterion; SABIC, Sample-size adjusted BIC.

*There is some controversy over estimating fit indices and RMSEA values with small numbers in degrees of freedom (Kenny et al., 2015) but the present models did not present that limitation*.

**Figure 4:**
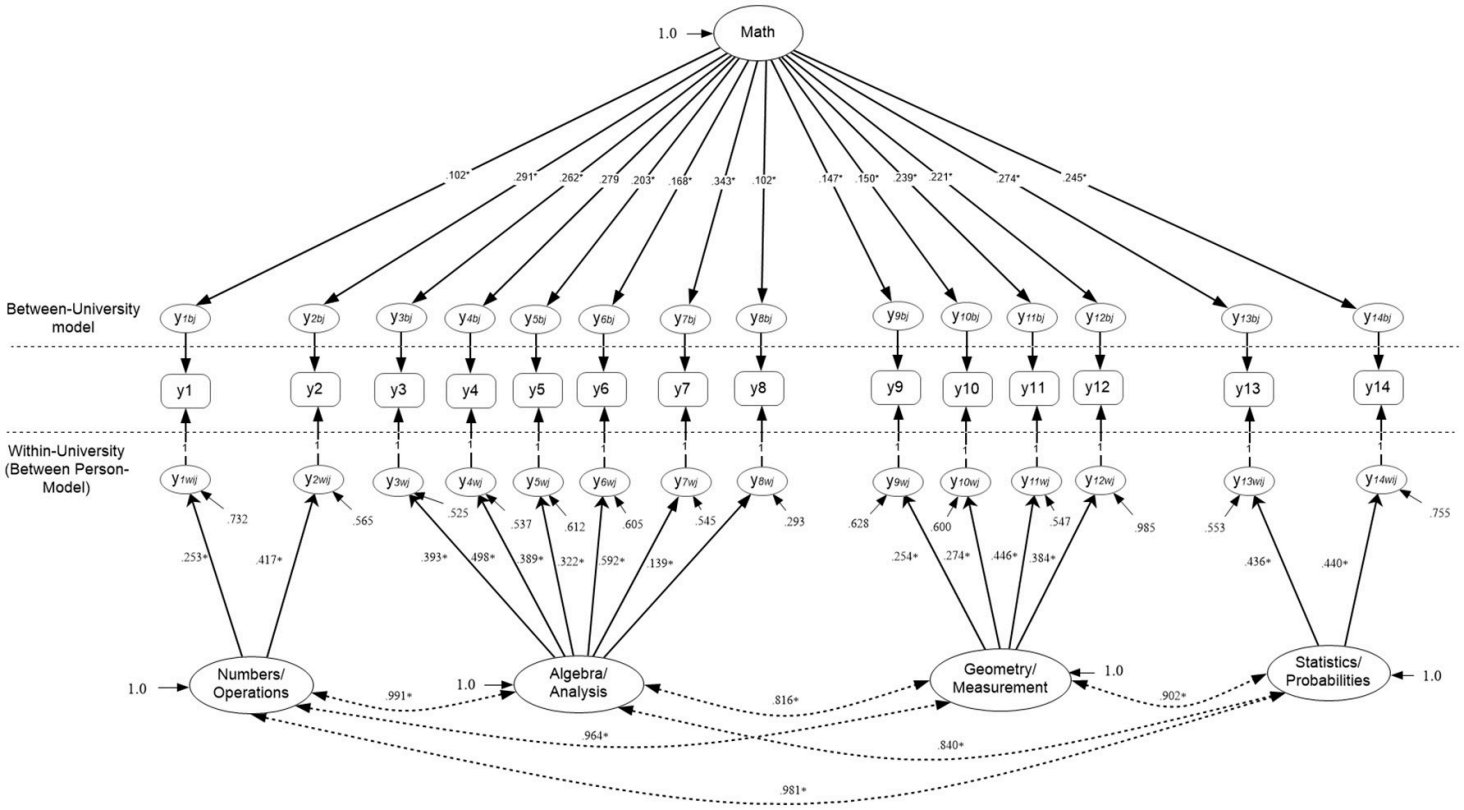
Optimal model for the measurement of math achievement at both the person and university levels of the analysis. All measurement and structural (between factor correlations) paths were significant at *p* < 0.01. Factor variances were standardized to unity for identification. Factor model indicators are based on parceling. The full sample of 2,881 participants contributed data in the evaluation of this model.

Table 3 initially shows the fit of the four competing models followed by chi-square difference tests in the case when models were nested, along with values from information criteria, for comparisons of non-nested models. The best model fit was associated with a one-factor model structure at the between level and a 4-factor correlated structure at the between level (4W1B) of the analysis (RMSEA < 0.001, CFI = 1.0, TLI = 1.0, SRMR_Within_ = 0.016, SRMR_Between_ = 0.037) with the chi-square test being non-significant [$\chi_{\left(148\right)}^{2}$ = 130.885, *p* = 0.841] suggesting “exact fit” between the specified model and the data (MacCallum et al., 1996). This 4W1B model was superior to the unidimensional model at both levels in the analysis by use of a chi-square difference test [Δ_Chi−square__(6)_ = 59.472, *p* ≤ 0.001]. In the comparison between the preferred 4W1B model and the 4-factor model at both levels (4W4B), the chi-square difference test was not significant. In the case of two models that one is not clearly superior we opt for the less complex model based on the principle of parsimony. However, when utilizing information criteria, it appears that the more complex model (i.e., 4W4B) was associated with larger AIC and BIC values, in excess of 10 units (AICDIF = −10.078, BICDIF = −49.694). Based on the work of Raftery, when AIC difference values exceed 10 units, there is strong evidence that one model is superior to the other. Thus, the 4W1B model appears to be the preferred choice with these data (see Figure 4). Further analyses to verify the validity of the proposed structure follow in the next section with a quantification of misfit per specified model at each level in the analysis.

### Verifying multilevel simple structures through estimating level-specific misfit: an application of the ryu and west (2009) partially saturated model methodology

Before concluding the optimal factor structure at any level in the analysis it was important to evaluate the level of misfit between competing models as a function of the information provided at that level only. The methodology has been described by Ryu and West (2009) as the partially saturated approach in that the level that is not tested is saturated so that it does not contribute any measurement error toward the overall fit of the model. Thus, when the 4-factor model was fitted to the data at the person level (within), with a saturated model at the between level, model fit was good [$\chi_{\left(71\right)}^{2}$ = 107.784, *p* = 0.003, RMSEA = 0.013, CFI = 0.992, TLI = 0.979]. At a second step, a unidimensional model was fit to the data and produced the following fit [$\chi_{\left(77\right)}^{2}$ = 166.446, *p* < 0.001, RMSEA = 0.020, CFI = 0.980, TLI = 0.953]. Because the two models are nested a chi-square difference test was utilized that was equal to the difference in chi-square units between the two models and was evaluated with the respective difference in the number of degrees of freedom. Results indicated that the difference chi-square statistic was equal to 58.662 units, which was significantly different from zero with 6 degrees of freedom (the critical value was 12.592 chi-square units). Thus, the 4-factor correlated model at the within level of the analysis provided superior model fit compared to the unidimensional structure and was associated with low amounts of measurement error.

A similar evaluation took place at the between level in the analysis through saturating the within level model. The fit of the 4-factor correlated model [$\chi_{\left(71\right)}^{2}$ = 18.680, *p* = 1.00, RMSEA < 0.001, CFI = 1.0, TLI = 1.0] was contrasted to that of the unidimensional structure at the university level [$\chi_{\left(77\right)}^{2}$ = 22.174, *p* = 1.0, RMSEA < 0.001, CFI = 1.0, TLI = 1.0]. Results pointed to accepting the null hypothesis that both models fit the data equally well. Consequently, due to parsimony, the 1-factor model was deemed the most appropriate structure at the university level (between person level).

### Testing for the discriminant validity of the optimal multilevel SEM model

One important hypothesis related to the discriminant validation of the mathematics measure as the between factor correlations were very high. To this end, we compared the freely estimated correlated factor model at the within level and saturated between [$\chi_{\left(71\right)}^{2}$ = 107.784, *p* = 0.003, RMSEA = 0.013, CFI = 0.992, TLI = 0.979] to a model in which the within factor relationships were constrained to be equal to 1.0 and the between model again saturated [$\chi_{\left(77\right)}^{2}$ = 166.446, *p* < 0.001, RMSEA = 0.02, CFI = 0.980, TLI = 0.953]. If this later model fits the data equally well compared to the 4-factor freely correlated model, then between factor correlations equal to 1.0 would represent plausible values. Consequently, discriminant validation would be lacking. When comparing the fit of the 4-factor correlated model with freely estimated between-factor correlations to that of the same simple structure but with fixed correlations to unity, results indicated significant misfit of the later as the difference chi-square value was equal to 58.662 with again a critical value of 12.592. Thus, a conclusion of discriminant validation was supported as the model with fixed correlations was statistically inferior to that of the 4-factor freely correlated model.

### Testing for measurement invariance and the presence of item bias due to gender: a multiple indicator multiple causes differential item functioning (DIF) analysis^11^

**Figure 5:**
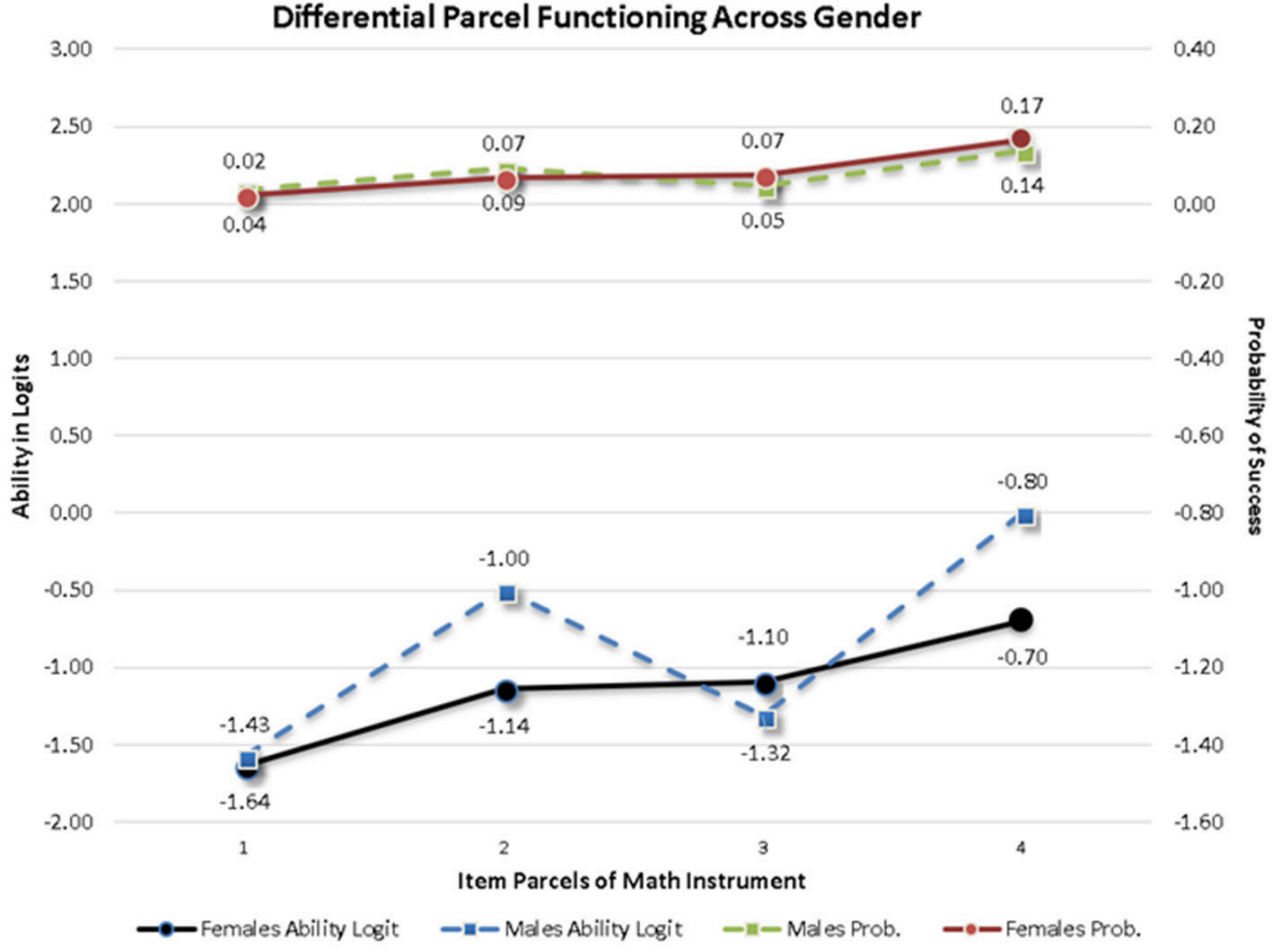
Item parcels showing significant Differential Item Functioning (DIF) across mathematics items. There were 4 out of the 14 item parcels. Bottom two lines show differences between males and females on the logit scale and those at the top of the graph at the probability scale. Differences are likely reflective of Type-I errors. Notably, significant DIF was observed on difficult items, thus, probability of success is low for both groups.

A MIMIC model was applied at the within (person) level to test the measurement invariance of the instrument across gender although alternative approaches based on multi-group modeling are also available (Kim and Cao, 2015). Based on recommendations by Muthén (1989a) the effects of the covariate and measurement non-invariance should be examined by constraining the effects of the covariate on the item parcels to be zero and through examining the misfit documented in the modification indices. After fitting this constrained model to the data, results indicated that item parcels 1, 3, 4, and 10 were associated with increases in chi-square values between 11.039 and 38.985 units, all significant given a critical threshold value of 10 chi-square units. Furthermore, a direct effect of gender on the first factor (Numbers/Operations) was significant and negative suggesting that females had lower scores than males on that factor. Inspection of the behavior of item parcel 1 that loaded onto the Numbers/Operations factor revealed that its factor loading was positive, thus, the expectation was that females would have higher scores compared to males on that item parcel. The covariate effect, however, was negative and significant suggesting that females actually had lower scores on that item parcel. That was evidence of measurement non-invariance for the first arithmetic item parcel across gender. The same exact effect was also observed with item parcel 3, the first item of the Algebra/Analysis factor pointing again to the presence of non-invariance due to gender. For item parcels 4 and 10, however, there was no significant effect on the factor mean pointing to an expectation that differential responding should not be expected across gender. Nevertheless, the effects of the covariate were significant and positive suggesting that females, had significantly elevated scores on those item parcels, indicating non-invariance or the presence of Differential Item Functioning (DIF). Figure 5 displays the difference across gender on both the logit (y-axis to the right) and the probability scale (y-axis to the left of the figure). As shown in Figure 5, differences that exceeded levels of significance were practically meaningless. For example, at the probability scale, success rates between 2 and 4% were significantly different from each other but likely represent miniscule differences using an effect size metric. The largest difference represented 3 percentage units. Furthermore, the pattern of findings was not consistent in that all four item parcels were favored by males only or females only suggesting a balance across gender that is likely reflective of random variation that exceeded levels of significance due to excessive levels of power of the z-test statistics. Consequently, a conclusion of measurement invariance was drawn, suggesting that the few significant observed discrepancies likely reflect Type-I errors due to the large sample size.

### Testing for level differences across gender using multilevel MIMIC model

Assuming measurement invariance across gender as per the previous section, with the few significant findings reflecting very low effect sizes and were rather an artifact of the large sample size, a latent means analysis was conducted using procedures described by Kim and Cao (2015). Consequently, the latent factor means of the four math constructs were regressed on a dummy gender variable. Results pointed to the presence of null effects across all constructs except the Numbers/Operations factor. The mean of females was −0.214 units lower compared to that of males on the respective construct (*z* = −2.791, *p* = 0.005).

### Multilevel structural equation modeling (MSEM) for the prediction of math achievement from type of university (old-new)

**Figure 6:**
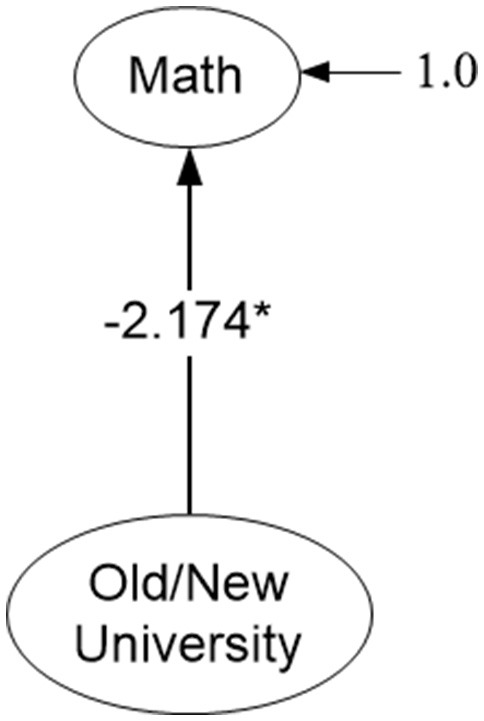
Effects of type of university (old and new establishments) on the math achievement of university students. Only latent variables and level-2 predictor are shown for parsimony. *p < 0.05, two-tailed test.

Following evaluation of the measurement models above, a last aim involved a structural model in which the latent factor mean of the unidimensional math structure at the between level of the analysis was regressed on the year the university was established (see Figure 6). Prior to testing this model, it was necessary to verify the measurement invariance of the model across old and new establishments. Consequently, a multi-group MSEM model was tested with age of university comprising the between-level grouping variable. Factor loadings and intercepts were constrained to be equivalent across type of universities and model fit was subsequently evaluated. Results indicated that imposing these constraints was associated with excellent model fit. Specifically, the overall chi-square test was non-significant suggesting “exact fit” [$\chi_{\left(336\right)}^{2}$ = 308.371, *p* = 0.858] and among fit indices both the CFI and TLI were equal to 1. The RMSEA was less than 0.001 and the misfit introduced by the different levels of the age of institution variable were very similar (168.773 and 139.597 chi-square units for old and new establishments, respectively). Thus, a conclusion of strict measurement invariance was supported, and generalized math competency was subsequently regressed on a dummy variable (year the university was established) coded with zero representing older institutions and with a value of 1, newly established institutions. Results indicated that there were significantly lower math achievement levels in students nested within newer establishments compared to older ones (*b*_Math_ = −2.174, *p* < 0.001).

## Discussion

The measurement of academic achievement has been predominantly examined with person level data which essentially fail to disaggregate the person variability from that of between person structures such as the university students belong to. Consequently, when using person-based estimates of achievement any influences due to university are confounded. The purpose of the present study was to model math achievement at both the person and university levels of the analyses in order to understand the optimal factor structure of math achievement using information from the factor model so that all available information regarding the measurement of math achievement is accounted for. Several salient findings emerged, which are presented in order of importance in the sections that follow.

The most important finding related to the fact that the simple structure of math achievement appears to be different when viewed under the lenses of the aggregated model (person level data) and under the differentiation as person level and university level data. Specifically, the aggregate data analysis supported a conclusion of an optimal univariate model for the measurement of math achievement and the multilevel structural equation model a conclusion favoring a unidimensional structure at the university level and a multi-factor model at the person level. This is an important consideration that affects both theory and measurement practice and utility. That is, a simple structure should be evaluated for fit at each level in the analysis and that conclusion should inform theory; also, those findings should inform measurement in that they should lead to simple structures with the least amount of measurement error so that subsequent phenomena (i.e., structural relations) would be modeled properly with the most appropriate measurement models for each level in the analysis. At the between university level, the correlation between constructs was high suggesting that math ability at the university level is driven by the overall capacity of the university, without showing domain specific effects. At the person level, differential performance (low-high achievement) across subspecialties was observed suggesting that individuals may have a preference and different level of skill in some math area (e.g., statistics) but less so in another (e.g., algebra). So, although at the university level, institutions were either good or not so good across math specialties, math achievement at the person level was governed by math subspecialty in that performance in one subject matter was unrelated to the performance in another subject matter (between factor correlations were at times zero). The findings at the person level are expected in that individuals should not necessarily be “equally” good across math specialties. The finding at the university level did not provide support to the hypothesis that there are different emphases within a university (defined by different quality staff and resources so that for example, a department within a university may emphasize statistics but less so, algebra and analysis). This apparently diverse simple structure observed at the university level compared to the aggregate data is surprising provided that the ICCs were not that large to warrant such a saliently different solution (Opdenakker and Van Damme, 2000).

The second most important finding relates to the ability of the MSEM model to understand the variability of university phenomena, after employing the most appropriate simple structure, using university level predictors, after first verifying measurement invariance. In the present study, the age of an institution was factored in, to understand math achievement with strict invariance being justified across old and new institutions. Results favored older institutions in that math achievement was significantly elevated. This finding agrees with previous data from e.g., UK university evaluations in 2016 in that older institutions had significantly higher ranking, research quality and intensity, better student to staff ratio, significantly higher allocation of funds, more facilities, higher honors, and higher completion rates (University League Tables and Rankings, 2017). Thus, the present analysis using the university as the unit of analysis and after evaluating the most optimal simple structure at the university level, allows for a proper evaluation of university departments, their degree of competency and production, which is primarily associated with funding from federal and/or state sources. Interestingly, in the U.S. the major research institutions (that are mostly older) seem to be penalized with regard to funding as the likely newer community colleges and non-research institutions seem to be receiving the largest share of their budgets from federal and state funds (Woodhouse, 2015). In the absence of the MSEM methodology, one could neither test for the most optimal model using the university as the level of analysis, nor would be able to predict how the age of the institution could contribute to achievement in math. Subsequent public policy decisions could then be adjusted for the present findings.

A third important finding related to the evaluation of within level predictors such as person demographic characteristics. In the present study the effects of gender were evaluated after first establishing partial measurement invariance of the 4-factor solution between males and females. Only four out of the 14 item parcels showed significant amounts of DIF, which, when evaluated using practical means appeared to be very small and, in that sense, insignificant. Consequently, measurement invariance was assumed and, in a MIMIC, structural model all latent factors were regressed onto the dummy gender variable. Results pointed to the existence of minimal differences across gender, with one significant effect observed for factor 1 (numbers/operations), with females having a significantly lower mean on that construct. Overall, these findings suggest that males and females are comparable in their levels of math achievement across math domains and contrasts earlier findings pointing to the existence of gender differences with females having lower aptitude in math compared to males across math domains (e.g., Régner et al., 2016).

The present study is also limited for several reasons. First, the most optimal simple structure was not consistently pursued through deleting item parcels or persons as the goal of the present study was not to purify and improve the instrument under study. Furthermore, disaggregation was the preferred method of analysis provided that the current measure was reflective rather than formative. Thus, we deferred from this approach of initially purifying the measure using aggregate data. A third limitation pertains to the fact that several intraclass coefficients were low, particularly since low ICC values (along with other factors) have been implicated with biased estimates of factor loadings (Muthén and Satorra, 1995; Hox and Maas, 2001; Wu and Kwok, 2012), non-convergence (Toland and De Ayala, 2005), and/or inadmissible estimates such as the presence of negative variance estimates (Li and Beretvas, 2013), leading to proposals for involving Bayesian estimation approaches (Depaoli and Clifton, 2015). In the present study, issues of non-convergence were not present suggesting that the large cluster sizes (mean cluster size = 247 participants) acted as a buffer to estimation problems (Preacher et al., 2011) along with creating item parcels as categorical data have been largely implicated with estimation problems and non-convergence (Yang-Wallentin et al., 2010). As Muthén (1991) pointed out, small ICC values are common in educational and psychological research, as well as small cluster sizes (e.g., with 5–20 participants, Mathisen et al., 2006), however, research has shown that ignoring ICCs as low as 0.02 lead to parameter inflation and a large number of Type-I errors (Murray and Hannan, 1990; Siddiqui et al., 1996; Baldwin et al., 2011). Another limitation pertains to the unbalanced samples in old and new establishments, which, may have affected the generalizability of the findings. Last, fit at the within level may suggest an overidentified model, which potentially creates problems with parameter estimation.

Nevertheless, the present study's novelty lies on the fact that proper measurement of many conceptual phenomena likely involves “nesting.” The use of the factor model as part of multilevel modeling further disattenuates measurement error and provides improved accuracy of person scores. The simultaneous modeling of the covariance structure at both levels in the analysis allows for a proper disaggregation of variances and covariances at each level. Under those lenses latent variable models are the most appropriate means for assessing construct validity and should be tested separately at each level in the analyses as needed in order to more accurately measure the constructs under study. However, despite the analytical benefits, as Stapleton et al. (2016) have noted, if theoretically speaking the interest is at the person level and the construct being measured also makes only sense to be assessed at that level only, modeling level-2 structures, may not be appropriate.

## Ethics statement

This study was carried out in accordance with the recommendations of the human experimentation committee of the National Center for Assessment in Higher Education.

## Author contributions

GS drafted the manuscript, run statistical analyses, created tables and figures, and monitored all aspects of the written product. IT contributed significantly to the write-up of the study, run some data analyses and created tables. He approved all aspects of the written product. AA-S collected the data, drafted parts of the data analysis section, proofread the entire manuscript and approved all parts of the written product.

### Conflict of interest statement

The authors declare that the research was conducted in the absence of any commercial or financial relationships that could be construed as a potential conflict of interest.

## Footnotes

^1^Throughout the manuscript, items and item parcels have been used interchangeably.

^2^Absolute in the sense that the best fitted model returns a value of zero.

^3^Incremental or relative in that the best fitted model returns a value of one and zero reflects the worst fitted model, usually defined as the independence model in which there is no relation between variables but means of the observed variables are estimated at their true value.

^4^Use of the standard independence model is inappropriate as it would yield spurious estimates of model fit by use of indices such as the CFI and other because model fit will include the saturated part of the model that provides no misfit to the overall evaluation.

^5^Interestingly, the 4-factor within and 4-factor between simple structure was equally appropriate to our preferred 4-factor within, 1-factor between structure but for reasons of parsimony was eventually not the preferred structure.

^6^Obviously support of the alternative hypothesis suggests that the misfit of the data-based model is greater than zero.

^7^This parametric bootstrap distribution was assumed to be normal.

^8^That is, ignoring for level-specific estimates of the measure's simple structure. Cluster membership was ignored.

^9^Moerbeek (2004) recommended that the larger the ICCs the more detrimental the effects are of disaggregation (i.e., ignoring the information at the macro level).

^10^The high between factor correlations warranted the need to test a hierarchical structure of math achievement at the within level. Unfortunately, there were problems in the estimation of the slope of the “Number and Operations” factor with the hierarchical entity. When fixing that slope to unity, model fit of the hierarchical model was deteriorated significantly. That is, for a difference of 3 degrees of freedom misfit introduced by the chi-square test equaled 62.438 points, which, for a critical value of 7.815 chi-square units was significant. Consequently, the hierarchical model did not provide a plausible model with the present data, mostly due to estimation problems so, although we cannot rule out this model, we cannot adopt it either.

^11^As in a DIF analysis with polytomous data. Item parcels had a maximum of 4 levels, thus DIF could be examined with item parcels.

## References

[B1] Adelman C. (1999). Answers in the Tool Box: Academic Intensity, Attendance Patterns, and Bachelor's Degree Attainment. Washington, DC: Government Printing Office; U.S. Department of Education, Office of Educational Research and Improvement.

[B2] Adelman C. (2003). Postsecondary Attainment, Attendance, Curriculum and Performance (NCES 2003-394). Washington, DC: National Center for Education Statistics; U. S. Department of Education, Institute of Education Science.;Government Printing Office.

[B3] Aghion P. Dewatripont M. Hoxby C. Mas-Colell A. Sapir A. (2010). The governance and performance of Universities: evidence from Europe and the US. Econo. Policy 25, 8–59. 10.1111/j.1468-0327.2009.00238.x

[B4] Ambady N. Shih M. Kim A. Pittinsky T. L. (2001). Stereotype susceptibility in children: effects of identity activation on quantitative performance. Psychol. Sci. 12, 385–390. 10.1111/1467-9280.00371 11554671

[B5] Bagozzi R. P. Heatherton T. F. (1994). A general approach to representing multifaceted personality constructs: application to state self-esteem. Struct. Equat. Model. 1, 35–67. 10.1080/10705519409539961

[B6] Baldwin S. A. Murray D. M. Shadish W. R. Pals S. L. Holland J. M. Abramowitz J. S.. (2011). Intraclass correlation associated with therapists: estimates and applications in planning psychotherapy research. Cogn. Behav. Ther. 40, 15–33. 10.1080/16506073.2010.520731 21337212 PMC3650614

[B7] Bandalos D. L. Finney S. J. (2001). “Item parceling issues in structural equation modeling,” in Advanced structural equation modeling: New Developments and Techniques, eds Marcoulides G. A. Schumacker R. E. (Hillsdale, NJ: Lawrence Erlbaum Associates, Inc), 269–296.

[B8] Barr A. B. (2015). Family socioeconomic status, family health, and changes in students' math achievement across high school: a mediational model. Soc. Sci. Med. 140, 27–34. 10.1016/j.socscimed.2015.06.028 26189011

[B9] Beauducel A. Herzberg P. Y. (2006). On the performance of maximum likelihood versus means and variance adjusted weighted least squares estimation in CFA. Struct. Equat. Model. Multidisciplinary J. 13, 186–203. 10.1207/s15328007sem1302_2

[B10] Brown T. A. (2015). Confirmatory Factor Analysis for Applied Research. New York, NY: Guilford.

[B11] Cvencek D. Kapur M. Meltzoff N. A. (2015). Math achievement, stereotypes, and math self-concepts among elementary-school students in Singapore. Learn. Instr. 39, 1–10. 10.1016/j.learninstruc.2015.04.002

[B12] Deci E. L. Ryan R. M. (1985). Intrinsic Motivation and Self-Determination in Human Behavior. New York, NY: Plenum.

[B13] Depaoli S. Clifton J. P. (2015). A Bayesian approach to multilevel structural equation modeling with continuous and dichotomous outcomes. Struct. Equat. Model. 22, 327–351. 10.1080/10705511.2014.937849

[B14] Dolan C. V. (1994). Factor analysis of variables with 2, 3, 5, and 7 response categories: a comparison of categorical variable estimators using simulated data. Br. J. Math. Stat. Psychol. 47, 309–326. 10.1111/j.2044-8317.1994.tb01039.x

[B15] Field A. (2000). Discovering Statistics Using Spss for Windows. Thousand Oaks, CL: Sage publications.

[B16] Field A. (2009). Discovering Statistics Using SPSS. London: SAGE.

[B17] Galdi S. Cadinu M. Tomasetto C. (2014). The roots of stereotype threat: when automatic associations disrupt girls' math performance. Child Dev. 85, 250–263. 10.1111/cdev.12128 23713580

[B18] Gallo J. J. Anthony J. C. Muthén B. O. (1994). Age differences in the symptoms of depression: a latent trait analysis. J. Gerontol. Psychol. Sci. 49, 251–264. 10.1093/geronj/49.6.P251 7963280

[B19] Gamoran A. (1992). The variable effects of high school tracking. Am. Sociol. Rev. 57, 812–828. 10.2307/2096125

[B20] Geldof G. J. Preacher K. J. Zyphur M. J. (2014). Reliability estimation in a multilevel confirmatory factor analysis framework. Psychol. Methods 19, 72–91. 10.1037/a0032138 23646988

[B21] Gorsuch R. (1983). Factor Analysis, 2nd Edn Hillsdale, NJ: Lawrence.

[B22] Gravetter F. Wallnau L. (2014). Essentials of Statistics for the Behavioral Sciences, 8th Edn Belmont, CA: Wadsworth.

[B23] Harley S. (2002). The impact of research selectivity on academic work and identity in UK Universities. Stud. Hr. Educ. 27, 187–205. 10.1080/03075070220119986b

[B24] Heck R. H. Thomas S. L. (2009). An Introduction to Multilevel Modeling Techniques, 2nd Edn. New York, NY: Routledge.

[B25] Heck R. H. Thomas S. L. (2015). An Introduction to Multilevel Modeling Techniques. New York, NY: Routledge.

[B26] Herndon J. S. Bembenutty H. (2014). In-school and social factors influencing learning among students enrolled in a disciplinary alternative school. Learn. Individ. Differ. 35, 49–55. 10.1016/j.lindif.2014.07.007

[B27] Hox J. (2002). Multilevel Analysis: Techniques and Applications. Mahwah, NJ: Lawrence.

[B28] Hox J. J. Maas C. J. M. (2001). The accuracy of multilevel structural equation modeling with pseudobalanced groups and small samples. Struct. Equat. Model. 8, 157–174. 10.1207/S15328007SEM0802_1

[B29] Hsu H. Y. Lin J. H. Kwok O. M. Acosta S. Willson V. (2016). The impact of intraclass correlation on the effectiveness of level-specific fit indices in multilevel structural equation modeling: a monte carlo study. Educ. Psychol. Meas. 77, 5–31. 10.1177/0013164416642823 29795901 PMC5965526

[B30] Huang F. L. Cornell D. G. Konold T. Meyer J. P. Lacey A. Nekvasil E. K.. (2015). Multilevel factor structure and concurrent validity of the teacher version of the authoritative school climate survey. J. Sch. Health 85, 843–851. 10.1111/josh.12340 26522173

[B31] Julian M. W. (2001). The consequences of ignoring multilevel data structures in nonhierarchical covariance modeling. Struct. Equat. Model. 8, 325–352. 10.1207/S15328007SEM0803_1

[B32] Kenny D. A. Kaniskan B. McCoach D. B. (2015). The performance of RMSEA in models with small degrees of freedom. Sociol. Methods Res. 44, 486–507. 10.1177/0049124114543236

[B33] Khattri N. Riley K. W. Kane M. B. (1997). Students at risk in poor, rural areas: a review of the research. J. Res. Rural Educ. 13, 79–100.

[B34] Kim E. S. Cao C. (2015). Testing group mean differences of latent variables in multilevel data using multiple-group multilevel CFA and multilevel MIMIC modeling. Multivar. Behav. Res. 50, 436–456. 10.1080/00273171.2015.1021447 26610156

[B35] Kreft I. G. G. de Leeuw J. (2004). Introducing Multilevel Modeling. Thousand Oaks, CA: Sage.

[B36] Kung H.-S. Lee Y.-C. (2016). Multidimensionality of parental involvement and children's mathematics achievement in Taiwan: mediating effect of math self-efficacy. Learn. Individ. Differ. 47, 266–273. 10.1016/j.lindif.2016.02.004

[B37] León J. Núnez L. J. Liew J. (2015). Self-determination and STEM education: effects of autonomy, motivation, and self-regulated learning on high school math achievement. Learn. Individ. Differ. 43, 156–163. 10.1016/j.lindif.2015.08.017

[B38] Li X. Beretvas S. N. (2013). Sample size limits for estimating upper level mediation models using multilevel SEM. Struct. Equat. Model. 20, 241–264. 10.1080/10705511.2013.769391

[B39] Longford N. T. Muthén B. O. (1992). Factor analysis for clustered observations. Psychometrika 57, 581–597.

[B40] Maas C. J. M. Hox J. J. (2005). Sufficient sample sizes for multilevel modeling. Methodology 1, 86–92. 10.1027/1614-2241.1.3.86

[B41] MacCallum R. C. Browne M. W. Sugawara H. M. (1996). Power analysis and determination of sample size for covariance structure modeling. Psychol. Methods 1, 130–149. 10.1037/1082-989X.1.2.130

[B42] Marsh H. W. (1990). The structure of academic self-concept: the Marsh/Shavelson model. J. Educ. Psychol. 82, 623–636. 10.1037/0022-0663.82.4.623

[B43] Marsh H. W. Hau K. T. Balla J. R. Grayson D. (1998). Is more ever too much: the number of indicators per factor in confirmatory factor analysis. Multivar. Behav. Res. 33, 181–220. 10.1207/s15327906mbr3302_1 26771883

[B44] Maruyama G. (2012). Assessing college readiness: should we be satisfied with ACT or other threshold scores? Educ. Res. 41, 252–261. 10.3102/0013189X12455095

[B45] Mathisen G. E. Torsheim T. Einarsen S. (2006). The team-level model of climate for innovation: a two-level confirmatory factor analysis. J. Occup. Organ. Psychol. 79, 23–35. 10.1348/096317905X52869

[B46] McCormack J. Propper C. Smith S. (2014). Herding cats? Management and university performance. Econ. J. 124, F534–F564. 10.1111/ecoj.12105

[B47] McDonald R. P. Goldstein H. (1989). Balanced and unbalanced designs for linear structural relations in two-level data. Br. J. Math. Stat. Psychol. 42, 215–232.

[B48] Mislevy R. (1986). Recent developments in the factor analysis of categorical variables. J. Edu. Stat. 11, 3–31. 10.3102/10769986011001003

[B49] Moerbeek M. (2004). The consequence of ignoring a level of nesting in multilevel analysis. Multivar. Behav. Res. 39, 129–149. 10.1207/s15327906mbr3901_5 26759936

[B50] Möller J. Pohlmann B. Köller O. Marsh H. W. (2009). Meta-analytic path analysis of the internal/external frame of reference model of academic achievement and academic self-concept. Rev. Educ. Res. 79, 1129–1167. 10.3102/0034654309337522

[B51] Murray D. M. Hannan P. J. (1990). Planning for the appropriate analysis in school-based drug-use prevention studies. J. Consult. Clin. Psychol. 58, 458–468. 2212183 10.1037//0022-006x.58.4.458

[B52] Muthén B. (1978). Contributions to factor analysis of dichotomous variables. Psychometrika 43, 551–560. 10.1007/BF02293813

[B53] Muthén B. (1989a). Latent variable modeling in heterogeneous populations. Psychometrika 54, 557–585.

[B54] Muthén B. (1989b). Latent variable models for dichotomous outcomes: analysis of data from the epidemiological catchment area program. Sociol. Methods Res. 18, 19–65.

[B55] Muthén B. O. (1991). Multilevel factor analysis of class and student achievement components. J. Educ. Measur. 28, 338–354.

[B56] Muthén B. O. Asparouhov T. (2009). “Growth mixture modeling: analysis with non-Gaussian random effects,” in Longitudinal Data Analysis, eds Fitzmaurice G. Davidian M. Verbeke G. Molenberghs G. (Boca Raton, FL: Chapman and Hall/CRC Press), 143–165.

[B57] Muthén B. O. Satorra A. (1995). Complex sample data in structural equation modeling. Sociol. Methodol. 25, 267–316.

[B58] Opdenakker M. C. Van Damme J. (2000). Effects of schools, teaching staff and classes on achievement and well-being in secondary education: similarities and differences between school outcomes. Sch. Eff. Sch. Improv. 11, 165–196. 10.1076/0924-3453(200006)11:2;1-Q;FT165

[B59] Panter A. T. Swygert K. A. Dahlstrom W. G. Tanaka J. S. (1997). Factor analytic approaches to personality item-level data. J. Pers. Assess. 68, 561–589. 10.1207/s15327752jpa6803_6 16372867

[B60] Pornprasertmanit S. Lee J. Preacher K. J. (2014). Ignoring clustering in confirmatory factor analysis: some consequences for model fit and standardized parameter estimates. Multivar. Behav. Res. 49, 518–543. 10.1080/00273171.2014.933762 26735356

[B61] Preacher K. J. Selig J. P. (2012). Advantages of monte carlo confidence intervals for indirect effects. Commun. Methods Meas. 6, 77–98. 10.1080/19312458.2012.679848

[B62] Preacher K. J. Zhang Z. Zyphur M. J. (2011). Alternative methods for assessing mediation in multilevel data: the advantages of multilevel SEM. Struct. Equat. Model. 18, 161–182. 10.1080/10705511.2011.557329

[B63] Rabe-Hesketh S. Skrondal A. Pickles A. (2004). Generalized multilevel structural equation modeling. Psychometrika 69, 167–190. 10.1007/BF02295939

[B64] Raftery A. E. (1995). Bayesian model selection in social research (with discussion). Sociol. Methodol. 25, 111–195.

[B65] Raudenbush S. W. Bryk A. S. (2002). Hierarchical Linear Models. Newbury Park, CA: Sage.

[B66] Raykov T. Marcoulides G. (2015). Intraclass correlation coefficients in hierarchical design studies with discrete response variables: a note on a direct interval estimation procedure. Educ. Psychol. Meas. 75, 1063–1070. 10.1177/0013164414564052 29795853 PMC5965596

[B67] Raykov T. (1997). Estimation of composite reliability for congeneric measures. Appl. Psychol. Meas. 21, 173–184. 10.1177/01466216970212006

[B68] Reeves B. E. (2015). The effects of opportunity to learn, family socioeconomic status, and friends on the rural math achievement gap in High School. Am. Behav. Sci. 56, 887–907. 10.1177/0002764212442357

[B69] Régner I. Selimbegović L. Pansu P. Monteil J.-M. Huguet P. (2016). Different sources of threat on math performance for girls and boys: the role of stereotypic and idiosyncratic knowledge. Front. Psychol. 7:637. 10.3389/fpsyg.2016.00637 27199863 PMC4850747

[B70] Ryu E. West S. G. (2009). Level-specific evaluation of model fit in multilevel structuralequation modeling. Struct. Equat. Model. 16, 583–601. 10.1080/10705510903203466

[B71] Shavelson R. J. Hubner J. J. Stanton G. C. (1976). Self-concept: validation of construct interpretations. Rev. Educ. Res. 46, 407–441. 10.3102/00346543046003407

[B72] Siddiqui O. Hedeker D. Flay B. R. Hu F. B. (1996). Intraclass correlation estimates in a school-based smoking prevention study: outcome and mediating variables, by sex and ethnicity. Am. J. Epidemiol. 144, 425–433. 10.1093/oxfordjournals.aje.a008945 8712201

[B73] Stapleton L. M. Yang J. S. Hancock G. R. (2016). Construct meaning in multilevel settings. J. Educ. Behav. Stat. 41, 481–520. 10.3102/1076998616646200

[B74] Tabachnick B. G. Fidell L. S. (2013). Using Multivariate Statistics. Boston, MA: Pearson.

[B75] Toland M. D. De Ayala R. J. (2005). A multilevel factor analysis of students' evaluations of teaching. Educ. Psychol. Meas. 65, 272–296. 10.1177/0013164404268667

[B76] Trochim W. M. Donnelly J. P. (2006). The Research Methods Knowledge base, 3rd Edn Cincinnati, OH: Atomic Dog.

[B77] U-Multirank (2014). Better With Age: Older Universities' Advantage in Research, Challenged by New Breed, Shows U-Multirank Study. Available online at: http://www.umultirank.org/

[B78] University League Tables Rankings (2017). The Complete University Guide. Available online at: http://www.thecompleteuniversityguide.co.uk/league-tables/rankings?v=wide

[B79] Vršnik Perše T. Kozina A. Rutar Leban T. (2010). Negative school factors and their influence on math and science achievement in TIMSS 2003. Educ. Stud. 37, 265–276. 10.1080/03055698.2010.506343

[B80] Werts C. E. Linn R. L. Jöreskog K. G. (1974). Intraclass reliability estimates: testing structural assumptions. Educ. Psychol. Meas. 34, 25–33. 10.1177/001316447403400104

[B81] Woodhouse K. (2015). Federal Spending has Overtaken State Spending as the Main Source of Public Funding in Higher Education. Available online at: https://www.insidehighered.com/news/2015/06/12/study-us-higher-education-receives-more-federal-state-governments

[B82] Wu J. Y. Kwok O. (2012). Using SEM to analyze complex survey data: a comparison between design-based single-level and model-based multilevel approaches. Struct. Equat. Model. 19, 16–35. 10.1080/10705511.2012.634703

[B83] Yang-Wallentin F. Jöreskog K. G. Luo H. (2010). Confirmatory factor analysis of ordinal variables with misspecified models. Struct. Equat. Model. 17, 392–423. 10.1080/10705511.2010.489003

[B84] Yuan K.-H. Bentler P. M. (2007). “Robust procedures in structural equation modeling,” in Handbook of Latent Variable and Related Models, ed Lee S.-Y. (North-Holland; Amsterdam), 367–397.

